# Proximate composition and mineral content of spices increasingly employed in the Mediterranean diet

**DOI:** 10.1017/jns.2023.52

**Published:** 2023-07-18

**Authors:** Ayesha S. Al Dhaheri, Dana Hasan Alkhatib, Abdul Jaleel, Maryam Naveed Muhammad Tariq, Jack Feehan, Vasso Apostolopoulos, Tareq M. Osaili, Maysm N. Mohamad, Leila Cheikh Ismail, Sheima T. Saleh, Lily Stojanovska

**Affiliations:** 1 Department of Nutrition and Health, College of Medicine and Health Sciences, United Arab Emirates University, Al Ain 15551, United Arab Emirates; 2 Department of Integrative Agriculture, College of Agriculture and Veterinary Medicine, United Arab Emirates University, Al Ain 15551, United Arab Emirates; 3 Institute for Health and Sport, Victoria University, Melbourne, VIC, Australia; 4 Immunology Program, Australian Institute of Musculoskeletal Science (AIMSS), Melbourne, VIC, Australia; 5 Department of Clinical Nutrition and Dietetics, College of Health Sciences, University of Sharjah, Sharjah 27272, United Arab Emirates; 6 Department of Nutrition and Food Technology, Faculty of Agriculture, Jordan University of Science and Technology, Irbid 22110, Jordan; 7 Nuffield Department of Women's & Reproductive Health, University of Oxford, Oxford OX1 2JD, UK

**Keywords:** Edible spices, Nutrition, Proximate analysis

## Abstract

The present study aimed to investigate the nutritional constituents of common market available spices in the United Arab Emirates. Seven commonly consumed spices namely, ginger (*Zingiber officinale*), cinnamon (*Cinnamomum verum*), black seed (*Nigella sativa*), fenugreek (*Trigonella foenum-graecum*), cardamom (*Elettaria cardamomum*), cloves (*Syzygium aromaticum*) and saffron (*Crocus sativus*) were obtained from local markets. Proximate analyses were performed according to AOAC procedures. Assessment of major (Ca, K, Mg, Na, P and S) and minor (Co, Cu, Fe, Mn and Zn) elements was conducted using inductively coupled plasma optical emission spectrometry (ICP-OES). Findings revealed varying macronutrient, micronutrient and mineral contents which are highly valuable for dietary purposes. The present study demonstrates that these edible spices could be used for nutritional support, due to their micro and macronutrient contents.

## Introduction

Spices have been extensively used in history as a component of daily diets of people from a wide variety of cultures and backgrounds. Spices are pungent or aromatic substances that are used for flavouring, colouring and preservation of food. Spices are all obtained from plants and can be fresh or dried seeds, kernels, bulbs, stalk, roots, bark, leave, pods or buds^(1)^. As a part of daily diets, spices could be a source of supplementary nutrients in addition to those obtained from whole foods.

Spices such as ginger (*Zingiber officinale*), cinnamon (*Cinnamomum verum*), black seed (*Nigella sativa*), fenugreek (*Trigonella foenum-graecum*), cardamom (*Elettaria cardamomum*), cloves (*Syzygium aromaticum*) and saffron (*Crocus sativus*) have been traditionally used particularly in the Indian subcontinent, Europe, Mediterranean and Arabian countries such as the United Arab Emirates (UAE)^(2)^. These spices are used for food preparation as aromatic spices and condiments. Spices have been studied for functional purposes in the contexts of diabetes, asthma, hypertension, inflammation, cough, bronchitis, headache, eczema, fever, dizziness and influenza^(3,4)^.

Even though spices are usually consumed in minute quantities, their contribution to the overall diet could be substantial if they were used more frequently. From a nutrition perspective, spices have significant roles in reducing lipids peroxidation during food processing and preparation, due to their inherent antioxidative activity. They also have antimicrobial activity, which is exploited by industrial food producers as natural preservatives^(5)^.

Ginger (*Zingiber officinale* Rosc.) belongs to the family Zingiberaceae and has been used in cooking and cultural medical practices for many years^(6)^. In addition to its macronutrient composition, ginger contains many different micronutrients including vitamins and minerals such as vitamin C, calcium, phosphorous, zinc, and iron and its polyphenol (tannins and flavonoids) makes it a good source of antioxidants. Cinnamon is obtained from the inner bark of plants in the genus *Cinnamomum* and has also been used as an edible and medicinal spice for centuries^(7)^. Such properties likely related to its vitamins and mineral content including potassium, copper, phosphate, zinc and iron as well as bioactive ingredients including cinnamaldehyde and cinnamic acid^(8)^.

Fenugreek (*Trigonella foenum-graecum*) is a legume seed belonging to the Fabaceae family and is widely produced and used in Mediterranean countries and Asia^(9)^. Fenugreek is primarily composed of carbohydrates and protein and to a lesser extent fibre and fat. Saffron (*Crocus sativus*), a carotenoid-rich spice belongs to the Iridaceous family, has been widely investigated due to its hypoglycaemic, hypolipidaemic and antioxidant properties^(3)^. Cardamom is a fibre-rich spice belonging to the Zingiberaceae family and is another commonly used spice that has been under extensive investigation in the last few years^(10)^. Furthermore, the plant *Nigella sativa* L. has edible seeds known as black seeds which contain several nutrients including copper, phosphate, zinc, iron and volatile and fixed oils providing benefits on top of its macronutrient content. Black seeds have been widely used in traditional medicine practices, but recent evidence has suggested antimicrobial, anticancer and antioxidant actions^(4)^. Although the studies on these spices are done with reference to many environmental and agricultural aspects, the studies of their biochemical constituents and mineral variations are rarely performed on market obtained samples. Therefore, the present study aimed to investigate the nutritional value of seven spices (ginger (*Zingiber officinale*), cinnamon (*Cinnamomum verum*), black seed (*Nigella sativa*), fenugreek (*Trigonella foenum-graecum*), cardamom (*Elettaria cardamomum*), cloves (*Syzygium aromaticum*) and saffron (*Crocus sativus*)) from a common market in the UAE.

## Materials and methods

### Sample preparation

Seven commonly consumed spices were purchased from a local market (Alyahar Market) in Al Ain, UAE. The selected spices included, ginger (*Zingiber officinale*), cinnamon (*Cinnamomum verum*), black seed (*Nigella sativa*), fenugreek (*Trigonella foenum-graecum*), cardamom (*Elettaria cardamomum*), cloves (*Syzygium aromaticum*) and saffron (*Crocus sativus*). Spices were purchased as a whole spice and were ground in the laboratory using a coffee and spice grinder machine (Moulinex Coffee Grinder, MC300161, France). Spice samples were prepared in triplicate for the proximate and micronutrient analyses.

### Proximate analysis

The seven spices were analysed chemically according to the Association of Official Analytical Chemists (AOAC) procedure^(11)^. Spices were analysed for their moisture, protein, fat, fibre and ash content according to the following procedures:

#### Moisture content determination

Moisture content of the seven spices were assessed by oven drying at 105°C for 2 h and placed in a desiccator to cool. One gram of each spice powder was weighed and spread uniformly in the aluminium dishes. A was used to dry the samples for a further 16 h at 105 ± 3°C which were then returned to the desiccator to cool down to room temperature. The equations used to calculate the percentage of total dry matter and total moisture are shown in Table 1^(11)^.

**Table 1.** Proximate analysis equations

Component	Equation
% Total dry matter	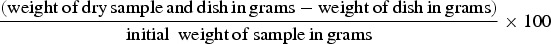
% Total moisture	100 − % Total dry matter
% Ash	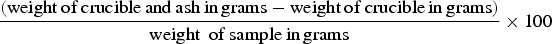
% Protein	% *Nitrogen* × Protein factor (6.25)
% Crude fat	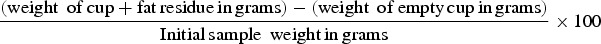
% Carbohydrate	100 − (% *Total* moisture + %*Ash* + %*Protein* + %*Crude* fat + % *Total* dietary fibre)

#### Ash determination

Labelled ashing crucibles were placed in a Mommert forced air drying oven (Schutzart DIN 400-50-IP20) at 500°C for 4 h, then placed in the desiccator to cool to room temperature. One gram of the sample was weighed in the crucibles and placed in a muffle furnace oven (Carbolite ELM, 11/6) at 500°C for 4 h. The crucibles were then allowed to cool to less than 200 °C. The equation used to calculate the percentage of ash is given in Table 1.

#### Protein content determination

The Kjeldahl method was applied to evaluate the nitrogen content in the different spice samples. The equation used to calculate the percentage of protein is given in Table 1.

#### Fat determination

Fat content was determined using Soxhlet extraction as recommended by the AOAC^(11)^. The equation used to determine percentage of crude fat is listed in Table 1^(11)^.

#### Fibre content determination

The ANKOMTDF Dietary Fiber Analyzer (Dietary Fiber Analyzer, ANKOM, Macedon, NY, USA) was used to measure the fibre content of each spice, using the AOAC 991.43 TDF method^(11)^.

#### Carbohydrate content determination

Carbohydrate content value was generated by the difference of the mean percentage values of moisture, ash, protein, lipids and dietary fibres shown in Table 1.

#### Energy calculation

The energy content of the spices samples was calculated according to Atwaters’ protocol^(12)^, as this method has been used previously and is considered suitable for the calculation of the energy content of the spices^(13)^. Energy content was calculated by the following equation:
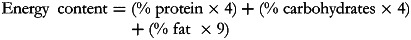


### Mineral content determination

The procedure for measuring minerals in spices by inductively coupled plasma optical emission spectrometer (ICP-OES) was followed for the micronutrient analysis of the spices^(14)^.

The CEM Mars 5 microwave digestion method (Mars5, CEM, Matthews, USA) was used for elements extraction from the spice samples. The digestion procedure was founded upon the USEPA 3015A guideline recommendations^(14)^. This microwave digestion method was designed to simulate extraction using conventional heating with nitric acid and hydrochloric acid. The spice samples were prepared by placing 0⋅50 g of sample into the microwave digestion vessels, with 10 ml of concentrated nitric acid and 2 ml hydrochloric acid. The vessels were capped and placed in the microwave digestion system. After the digestion and cooling of the samples to room temperature, de-ionised water was added to the sample solution to reach 50 ml before being aspirated through a nebulizer. The resulting solution was transported to a plasma torch for excitation.

Element-specific emission spectra were produced by radiofrequency inductively coupled plasma. The spectra were detached by a grating spectrometer, and intensities of the line spectra were monitored at specific wavelengths by a charged coupled detector. A fitted background correction was used to correct the blank signal and matrix effect. Background correction was not required in case of line broadening where a background correction measurement would degrade the analytical result. Emission spectra for each element were recorded as follows: Ca – 317⋅93 nm, K – 766⋅51 nm, Mg – 285⋅21 nm, Na – 588⋅99 nm, P – 177⋅43 nm, S – 180⋅67 nm, Co – 238⋅89 nm, Cu – 324⋅75 nm, Fe – 259⋅94 nm, Mn – 257⋅61 and Zn – 213⋅86 nm. Spectral, physical and chemical interferences were avoided through use of ionisation buffers, intra-experimental validation of solutions and automated spectral resolution. The machine used for the analysis was an ICP-OES (710-ES, Varian, USA).

### Statistical analysis

Statistical Package for Social Sciences (SPSS) version 23.0 (IBM Corp, Armonk, NY, USA) for windows was used for the analysis of the nutrient composition data. Kruskal–Wallis tests were used for comparison of measurements of macronutrients and micronutrients of the spices due to the lack of normality assumption of ANOVA. Statistical significances were considered with *P* < 0⋅05.

## Results

### Proximate analysis

Locally consumed spices were analysed chemically according to the AOAC. The proximate analysis data were expressed as mean and standard derivations (Table 2).

**Table 2.** Proximate analysis of the seven spices (mean ± standard deviation)

	Ginger	Cinnamon	Black seed	Fenugreek	Cardamom	Clove	Saffron
Moisture (g/100 g)	8⋅11 ± 0⋅26	8⋅24 ± 0⋅20	6⋅40 ± 0⋅13	7⋅33 ± 0⋅16	7⋅52 ± 0⋅09	7⋅86 ± 0⋅04	8⋅92 ± 0⋅19
Protein (g/100 g)	10⋅51 ± 0⋅26	3⋅50 ± 0⋅02	18⋅97 ± 0⋅13	24⋅99 ± 0⋅04	10⋅67 ± 0⋅08	6⋅96 ± 0⋅04	11⋅33 ± 0⋅05
Fat (g/100 g)	3⋅13 ± 0⋅20	0⋅55 ± 0⋅05	36⋅21 ± 0⋅11	4⋅36 ± 0⋅09	4⋅40 ± 0⋅27	5⋅04 ± 0⋅12	4⋅40 ± 0⋅07
Ash (g/100 g)	5⋅63 ± 0⋅04	4⋅60 ± 0⋅17	4⋅20 ± 0⋅05	0⋅02 ± 0⋅00	0⋅04 ± 0⋅00	5⋅68 ± 0⋅23	0⋅02 ± 0⋅00
Fibre (g/100 g)	3⋅01 ± 0⋅05	45⋅40 ± 0⋅89	20⋅67 ± 0⋅59	29⋅87 ± 0⋅78	30⋅13 ± 1⋅50	29⋅97 ± 1⋅99	12⋅23 ± 1⋅31
Carbohydrate (g/100 g)	69⋅61 ± 0⋅21	37⋅69 ± 0⋅49	13⋅55 ± 0⋅54	33⋅43 ± 0⋅61	47⋅23 ± 1⋅12	44⋅50 ± 1⋅81	63⋅10 ± 1⋅46
Energy (kcal)	348⋅65 ± 2⋅00	169⋅78 ± 2⋅36	455⋅98 ± 2⋅80	272⋅95 ± 2⋅99	271⋅20 ± 7⋅11	251⋅19 ± 7⋅23	337⋅32 ± 6⋅20

Data are expressed as g/100 g of whole dried spice powder.

Moisture content ranged from 6⋅40 g/100 g for black seed to 8⋅92 g/100 g for saffron. Moisture content is one of chief variables for assessing shelf life and our results showed lowest moisture content of black seed, suggesting increased shelf life when compared to other spices. Exposure of spices to a dry environment will result in reduced moisture content. However, they can absorb moisture again when exposed to humid environments, resulting in increased moisture content. The whole black seeds have low moisture content, but when ground, potentially absorb moisture from atmosphere and the water content increases, being greater than whole seeds, hence, moisture content increases with a greater surface area of the sample. Protein content was lowest in cinnamon at 3⋅50 g/100 g and highest in fenugreek at 24⋅99 g/100 g. Fat content was highest in black seed powder reaching 36⋅21 g/100 g, while it was lowest in cinnamon powder 0⋅05 g/100 g. Ash content varied from 0⋅02 g/100 g for fenugreek and saffron powder to 5⋅68 g/100 g for clove powder.

Cinnamon powder had the highest fibre level at 45⋅40 g/100 g, whereas ginger powder had the lowest level at 3⋅01 g/100 g. On the other hand, carbohydrate content ranged from 13⋅55 g/100 g for black seed powder to 69⋅61 g/100 g for ginger powder.

The moisture content of ginger was lower than earlier reports^(15)^, but higher than what was reported by Adeyeye *et al.*^(16)^. Fat (3⋅13 g/100 g) and ash (5⋅63 g/100 g) content were consistent with the previous studies, while fibre content (3⋅01 g/100 g) was significantly lower than previously reported^(15,17)^. Conversely, protein and carbohydrate content reported by Ereifej *et al.*^(17)^, Prakash *et al.*^(15)^ and Adeyeye *et al.*^(16)^ were lower than the present study findings. Protein content of cinnamon in our study was similar to that reported by Gul *et al.*^(18)^. While ash content of the present study (4⋅6 g/100 g) was similar to the ash content that Ereifej *et al.*^(17)^. In contrast, energy result reported by Gul *et al.*^(18)^ was significantly higher than the energy results of the present study due to a higher fat content. Moisture, protein, fat and ash content of the black seed in the present study are in consistent with the findings of previous studies^(19,20)^. Fibre content of the present study for black seed was higher than those reported previously, while carbohydrate content was lower than that reported in the literature^(19,20)^.

Naidu *et al.*^(21)^ reported a higher moisture content of fenugreek than the findings of the present study, while Al-Jasass *et al.*^(22)^ and El Nasri *et al*.^(23)^ reported similar moisture content to the present study findings. Protein content (24⋅9 g/100 g) was consistent with the study results of El Nasri *et al.*^(23)^ and Naidu *et al.*^(21)^ and higher than Al-Jasass *et al.*^(22)^ Cardamom moisture and ash content results were lower than what was reported by Ereifej *et al.*^(17)^, Pruthi *et al.*^(24)^ and Singh *et al.*^(25)^. While protein content of this study (10⋅67 g/100 g) was consistent with the results of Ereifej *et al.*^(17)^ and Singh *et al.*^(25)^, and higher than what Pruthi *et al.*^(24)^. Ereifej *et al.*^(17)^ reported a higher content of ash and fibre, while other studies^(24,25)^ reported a lower fibre content and a higher ash content. Carbohydrate content was higher than what was reported in literature^(23–25)^.

Earlier reports^(16,22)^ confirmed a similar protein content for cloves when compared to the present study findings (6⋅96 g/100 g). Moisture content of cloves in previous studies varied from 7⋅44 g/100 g for Al-Jasass *et al.*^(22)^ to 16⋅4 g/100 g for Ereifej *et al.*^(17)^. Our result was on the lower limit (7⋅87 g/100 g) and was consistent with Al-Jasass *et al.* findings^(22)^. Carbohydrate content of cloves (44⋅5 g/100 g) was lower than what previously reported^(16,22)^. In contrast, Ereifej *et al.*^(17)^ reported lower carbohydrate findings than the present study (31⋅3 g/100 g).

Saffron's moisture, fat and ash content (8⋅92 g/100 g, 4⋅4 g/100 g and 0⋅02 g/100 g, respectively) were lower than what Mohamadi *et al.*, Srivastava *et al.* and Fahim *et al.* reported^(26,27)^. Fibre and carbohydrate content (12⋅23 g/100 g and 63⋅1 g/100 g, respectively) were higher than what reported earlier by various researchers^(26,27)^.

The differences in nutrient composition of the spices reported is speculated to be due to the different soil and geographical locations of the spice plants, and due to the difference in environmental conditions, which influences the nutrient composition^(15,16)^. Moreover, different grinding and storing techniques have proved to have a major effect on the nutrient composition of the spices^(28)^. As such, the nutrient content of five different sizes of ginger powder particles that were produced using a micronizer machine, showed that the protein content increased significantly when the size of ginger powder particles decreased^(15)^. In an attempt to understand the differences in the nutrient composition between the spices with previous studies and the current research findings, it is believed that the superfine grinding of dried spices producing narrow and uniform particle size, increases the surface area and therefore increases the amount available for analysis^(29)^. In the present study, dried whole spices were grinded using a coffee grinding machine, with large particle-sized powders. Grinders can have different intensity levels, different blades and different durations. Therefore, different particle sizes could be produced using different grinders, hence, different nutrient composition findings as well^(29)^.

### Micronutrients composition analysis

#### Major elements

Results of the chemical analysis of the spices showed that these spices contain major elements in significant amount. As such, cinnamon had the highest calcium content (1414⋅82 mg/100 g and 141 %/100 g of RDA), while ginger had the lowest calcium content (125⋅21 mg/100 g and 12⋅5 %/100 g of RDA) (Table 3). Potassium content ranged from 460⋅78 mg/100 g (9⋅8 %/100 g of RDA) for cinnamon to 1125⋅91 mg/100 g (23⋅6 %/100 g of RDA) for saffron. Magnesium content ranged from 42⋅42 mg/100 g (10⋅1 %/100 g of RDA) for cinnamon to 375⋅71 mg/100 g (89⋅5 %/100 g of RDA) for cloves. On the other hand, sodium content was the lowest in cinnamon, cloves and saffron. Phosphorous ranged from 45⋅81 mg/100 g (6⋅5 %/100 g of RDA) for cinnamon to 675⋅52 mg/100 g (96⋅5 %/100 g of RDA) for ginger. Sulphur content did not exceed 310⋅58 mg/100 g (no established RDA) (black seed) in any of the analysed spices.

**Table 3.** Major elements composition of the spices (mean ± standard deviation)

Spice	Ca (mg/100 g)	K (mg/100 g)	Mg (mg/100 g)	Na (mg/100 g)	P (mg/100 g)	S (mg/100 g)
Ginger	125⋅21 ± 0⋅12	1071⋅13 ± 40⋅05	271⋅35 ± 2⋅3⋅00	69⋅80 ± 0⋅15	675⋅52 ± 4⋅84	176⋅40 ± 1⋅36
Cinnamon	1414⋅82 ± 8⋅11	460⋅78 ± 26⋅68	42⋅42 ± 0⋅15	1⋅74 ± 0⋅01	45⋅81 ± 0⋅53	76⋅13 ± 0⋅16
Black seed	578⋅24 ± 10⋅03	633⋅3 ± 54⋅60	263⋅60 ± 2⋅81	23⋅62 ± 0⋅35	649⋅15 ± 8⋅61	310⋅58 ± 4⋅84
Fenugreek	203⋅74 ± 2⋅95	853⋅95 ± 89⋅86	139⋅68 ± 2⋅95	35⋅13 ± 0⋅74	399⋅26 ± 6⋅68	237⋅91 ± 3⋅80
Cardamom	619⋅71 ± 20⋅55	873⋅30 ± 24⋅91	266⋅60 ± 12⋅41	316⋅79 ± 14⋅74	84⋅83 ± 4⋅12	130⋅54 ± 6⋅61
Clove	354⋅58 ± 3⋅50	1485⋅28 ± 72⋅12	375⋅71 ± 3⋅33	2⋅26 ± 0⋅01	125⋅84 ± 0⋅81	91⋅72 ± 0⋅15
Saffron	175⋅85 ± 2⋅85	1125⋅91 ± 13⋅62	222⋅32 ± 3⋅38	8⋅82 ± 0⋅18	391⋅83 ± 5⋅97	237⋅39 ± 3⋅63

Ca, calcium; K, potassium; Mg, magnesium; Na, sodium; P, phosphorous; S, sulphur.

Data are expressed as mg/100 g of whole dried spice powder.

#### Trace elements

Cobalt, copper, iron, manganese and zinc are trace minerals that play a major role in metabolism. The seven chosen spices were assessed for their trace mineral content in the current research study (Table 4). Trace minerals were found in smaller amount when compared to major minerals as shown in Table 3. None of the trace minerals exceeded 640 mg/100 g. Moreover, cobalt level was the highest in ginger while copper was the highest in black seed. Iron levels ranged from 624⋅77 mg/100 g (>7800 %/100 g of RDA for males and >3400 %/100 g of RDA for females) for saffron to 90⋅24 mg/100 g (1128 %/100 g RDA for males and 501⋅3 %/100 g of RDA for females) for cinnamon powder. Manganese content was the highest in clove powder (360⋅85 mg/100 g, 12 800–20 000 %/100 g of RDA [male–female]) and the lowest in fenugreek (23⋅90 mg/100 g, 1039–1327⋅8 %/100 g of RDA [male–female]), while zinc content did not exceed 56⋅24 mg/100 g (black seed powder) (511⋅3–703 %/100 g of RDA [male–female]) in any of the spice powders.

**Table 4.** Trace elements composition of the spices (mean ± standard deviation)

Spice	Co (mg/100 g)	Cu (mg/100 g)	Fe (mg/100 g)	Mn (mg/100 g)	Zn (mg/100 g)
Ginger	4⋅52 ± 0⋅09	0⋅28 ± 0⋅01	149⋅05 ± 1⋅15	230⋅65 ± 0⋅05	14⋅17 ± 0⋅15
Cinnamon	0⋅28 ± 0⋅10	2⋅70 ± 0⋅03	90⋅24 ± 0⋅36	171⋅52 ± 2⋅37	12⋅36 ± 0⋅10
Black seed	0⋅25 ± 0⋅01	16⋅52 ± 0⋅18	110⋅87 ± 1⋅11	30⋅64 ± 0⋅31	56⋅24 ± 0⋅78
Fenugreek	1⋅03 ± 0⋅06	9⋅28 ± 0⋅12	343⋅81 ± 2⋅60	23⋅90 ± 0⋅19	34⋅29 ± 1⋅00
Cardamom	0⋅52 ± 0⋅02	5⋅20 ± 0⋅07	421⋅37 ± 12⋅63	182⋅66 ± 5⋅46	12⋅98 ± 0⋅82
Clove	0⋅25 ± 0⋅06	7⋅06 ± 0⋅08	114⋅54 ± 0⋅89	360⋅85 ± 4⋅19	46⋅70 ± 0⋅62
Saffron	0⋅76 ± 0⋅02	9⋅85 ± 0⋅09	624⋅77 ± 7⋅43	32⋅05 ± 0⋅45	43⋅58 ± 1⋅33

Data are expressed as mg/100 g.

Co, cobalt; Cu, copper; Fe, iron; Mn, manganese; Zn, zinc.

## Discussion

The results of the present study were not in agreement with earlier studies^(30,31)^, which reported different mineral contents in ginger powder. In addition, the study of Prakash *et al.*^(15)^ showed that ginger powder contained 9⋅41 mg/100 g iron, 104⋅02 mg/100 g calcium and 204⋅02 mg/100 g phosphorous, of which calcium content was in accord to the present study.

An earlier study published by Gopalan *et al.*^(32)^ noted that spices have the following amounts of calcium, phosphorous and iron (mg/100 g) respectively: cardamom (229, 130 and 160), dried clove (740, 100 and 11⋅7), fenugreek (160, 370 and 6⋅5) and fresh ginger (20, 60 and 3⋅5). Chinese cinnamon was found to have the highest calcium content among the other identified elements, (Ca: 1157⋅36 mg/100 g, Mg: 74⋅89 mg/100 g and P: 66⋅31 mg/100 g, respectively); none of the minerals results of our study were consistent with these findings^(33)^. In a study by Khan *et al.*, it was reported that the manganese content for cinnamon, cardamom and cloves to be 879⋅8, 758⋅1 and 649⋅9 μg/g, respectively^(34)^, which are not in agreement to our findings.

Additionally, Maghrabi *et al.*^(35)^ analysed the commonly used spices in Saudi Arabia, including fenugreek and black seed and the results were not in agreement with our findings. On the other hand, Al-Jassir *et al.*^(36)^ indicated that potassium, phosphorus, sodium and iron levels to be the major elements present in black seed powder. While zinc, calcium, magnesium, manganese and copper were found at minor amounts. However, lead, cadmium and arsenic were not detected in the seeds as the study results are not consistent with the present study.

Mineral analysis of black seed reported by Cheikh-Rouhou *et al.*^(20)^ are in accordance with the present study in term of calcium and sodium content (5⋅75 mg/100 g and 20⋅4 mg/100 g, respectively), while the other mineral content values vary widely.

The mineral composition analysis of clove by earlier studies^(16,22)^ were not in agreement with the current findings. Adeyeye *et al.* reported remarkably higher levels of phosphorous, calcium and sodium (546 mg/100 g, 400 mg/100 g and 60 mg/100 g, respectively)^(16)^, while Al-Jasass *et al.* reported lower mineral content of clove powder in all minerals possibly due to the usage of different analytical techniques^(22)^. In regards to fenugreek, lower levels of all minerals content were reported by Al-Jasass *et al.*^(22)^, and higher levels reported by Naidu *et al*.^(21)^ when compared to the present study. Saffron, widely used in Iranian dishes, is a good source of potassium, magnesium, sodium, calcium, zinc, iron, copper and manganese^(37)^. Mineral levels fluctuate with species, and the difference in mineral content may increase due to the different analytical methods used, as well as this might be owing to the differences in the spice's origins^(38)^.

The differences in macronutrient composition of each of the spices reported are attributed to the different soil and geographical locations of the source of planting and growing plants, and due to the difference in environmental conditions, which influences the nutrient composition^(39–41)^. Different grinding and storing techniques have proven to have a major effect on the spice's nutrient composition^(42)^. Additionally, mineral levels fluctuate within species, as well as the analytical methods used for analysis. There is a general scarcity of literature that deals with these types of internal composition of spices, hence comparison between similar spices were difficult. Therefore, further studies are required to determine the nutrient content of sugar in the spices.

## Conclusions

Ginger, cinnamon, black seed, fenugreek, cardamom, clove and saffron were analysed in this study. Findings show that these spices have different micronutrient, macronutrient, mineral and lipid content. While each of these spices provides aroma and improves the taste of food, they are interestingly comprised of a wide range of diverse and valuable nutrients. Considering their well-documented medicinal properties and nutritional components, these spices or their active compounds could be commercially exploited for its application in therapeutic drugs or nutritional supplements providing health benefits. Keeping in view the potential of these edible spices, considerable efforts should be taken to encourage researchers to explore and develop necessary strategies for future preclinical and clinical research on these promising therapeutic leads.
